# The Role of Protein Disorder in Nuclear Transport and in Its Subversion by Viruses

**DOI:** 10.3390/cells9122654

**Published:** 2020-12-10

**Authors:** Jacinta M. Wubben, Sarah C. Atkinson, Natalie A. Borg

**Affiliations:** 1 Chronic Infectious and Inflammatory Diseases Research, School of Health and Biomedical Sciences, RMIT University, Bundoora, VIC 3083, Australia; jacinta.wubben@monash.edu (J.M.W.); sarah.atkinson@monash.edu (S.C.A.); 2 Infection and Immunity Program, Monash Biomedicine Discovery Institute and Department of Biochemistry and Molecular Biology, Monash University, Clayton, VIC 3800, Australia

**Keywords:** protein intrinsic disorder, nuclear transport receptors, nuclear export sequence, nuclear import sequence, nucleoporins, viral infection, nuclear import inhibitors, nuclear export inhibitors

## Abstract

The transport of host proteins into and out of the nucleus is key to host function. However, nuclear transport is restricted by nuclear pores that perforate the nuclear envelope. Protein intrinsic disorder is an inherent feature of this selective transport barrier and is also a feature of the nuclear transport receptors that facilitate the active nuclear transport of cargo, and the nuclear transport signals on the cargo itself. Furthermore, intrinsic disorder is an inherent feature of viral proteins and viral strategies to disrupt host nucleocytoplasmic transport to benefit their replication. In this review, we highlight the role that intrinsic disorder plays in the nuclear transport of host and viral proteins. We also describe viral subversion mechanisms of the host nuclear transport machinery in which intrinsic disorder is a feature. Finally, we discuss nuclear import and export as therapeutic targets for viral infectious disease.

## 1. Intrinsically Disordered Proteins Have Unique Features

For proteins to function they must have some degree of flexibility. At one end of the flexibility spectrum are ordered, globular proteins, which have a single well-defined structure stabilised by intramolecular interactions. At the other end of the spectrum are intrinsically disordered proteins (IDPs) that lack stable secondary and tertiary structure in solution and are consequently highly flexible and dynamic [1,2,3]. Proteins can also contain a combination of both ordered, and intrinsically disordered regions (IDRs). IDRs/IDPs represent a heterogenous conformation of structural ensembles that are capable of rapidly switching conformations in response to environmental changes. This structural heterogeneity is attributed to their biased amino acid composition, being rich in charged and polar amino acids, but poor in hydrophobic amino acids that would typically remain buried and stabilise the folded state.

Despite their lack of a well-defined structure, IDPs remain functional, and are typically involved in multiple protein-protein interactions, yet they maintain specificity [4]. Their low-complexity sequences are typically interspaced with short loosely structured regions, or molecular recognition elements, that serve as interaction sites for binding physiological partners, which may themselves be IDPs, globular/folded proteins or proteins containing both ordered and disordered regions. Upon binding their partner IDPs can obtain structure by undergoing a disorder-to-order transition; however, the extent of this transition can vary. The resulting complex may adopt a well-defined structure [5], or may be “fuzzy” and retain varying degrees of residual disorder [1,2]. Therefore, complexes involving IDPs can have variable binding properties and kinetics [6].

The plasticity of IDRs/IDPs renders them functionally versatile proteins, capable of interacting with multiple partners [7], and their extended nature affords them binding accessibility/exposure. Their extended nature is likely why IDPs are frequently targeted by post-translational modifications, such as phosphorylation [8] (see Section 8.2.4.), which in turn contribute to their sensitivity to physiological stimuli. Due to their versatility, IDRs/IDPs play many important roles within living cells, including a prominent role in the active transport of molecules between the nucleus and the cytoplasm [9].

## 2. Active Transport of Molecules across the Nuclear Envelope

The nuclear and cytoplasmic compartments of the cell are separated by an envelope consisting of a double lipid bilayer that is perforated with pores known as nuclear pore complexes (NPCs). The NPC functions as a selective permeability barrier that allows the transit of some molecules and prevents the passage of others. Generally, proteins smaller than ~40 kDa can passively diffuse through the NPC in a timely manner, and while larger cargoes can also undergo passive diffusion, their translocation efficiency is markedly reduced [10]. Alternatively, larger cargoes may be actively transported through the NPC when accompanied by nuclear transport receptors. Nuclear transport receptors, which are members of the karyopherin-β family, include nuclear import receptors (importins), nuclear export receptors (exportins), and bi-directional transporters [11]. Importins and exportins typically recognise protein cargo possessing nuclear localisation signals (NLSs) or nuclear export signals (NESs), respectively; however, cargo lacking these signals may piggyback onto others that contain the signals to enter or exit the nucleus [12,13]. In humans there are around 20 nuclear transport receptors [14], but here we focus on classical nuclear import and export via the importin α/β and exportin-1 nuclear transport receptors, respectively.

### 2.1. Classical Nuclear Import

**Figure 1:**
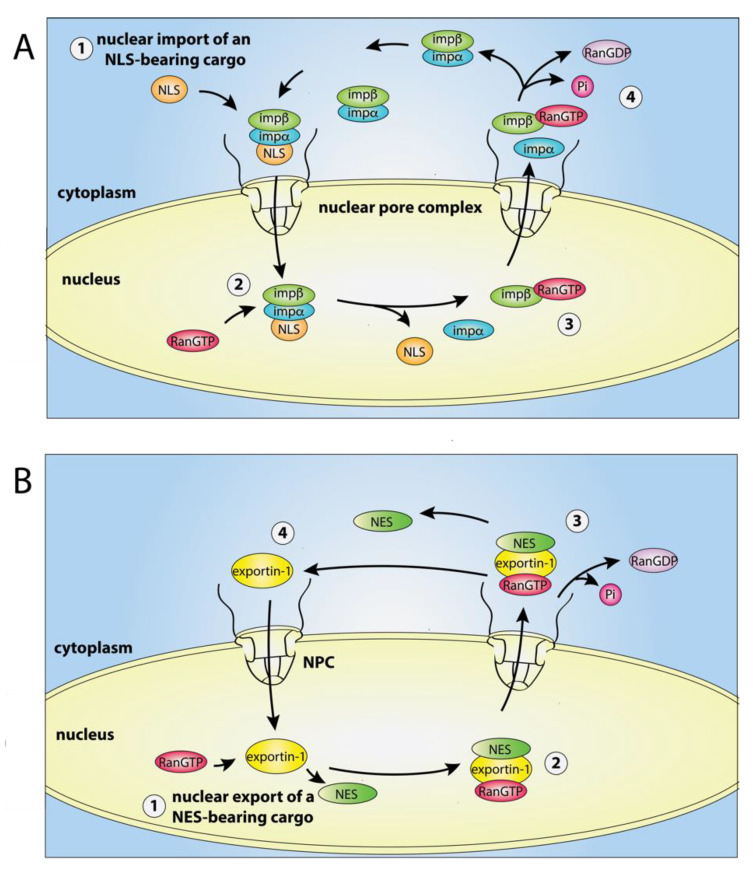
The nuclear import–export cycle. (**A**) Nuclear import of a nuclear localisation signal (NLS)-bearing cargo (orange) by importin α/β (imp α/β). Protein cargo in the cytosol bind the nuclear import receptor importin α/β for transport through the nuclear pore complex (NPC) to the nucleus. Following import, guanosine triphosphate (GTP)-bound Ran (RanGTP) binding to importin-β causes the release of the cargo in the cytoplasm and the recycling of importin-α and importin-β-RanGTP. (**B**) Nuclear export of a nuclear export signal (NES)-bearing cargo (green) by exportin-1. RanGTP binds to exportin-1 in the nucleus to enable the exportin-1-RanGTP-cargo ternary complex to be formed and exported to the cytoplasm. The ternary complex is disassembled following the hydrolysis of RanGTP to RanGTP (guanosine diphosphate (GDP)-bound Ran).

Whilst the prototypical importin, importin-β, can bind and import NLS-bearing cargo into the nucleus independently, it more commonly forms heterodimers with adaptors such as importin-α [15]. In this case, the importin α/β heterodimer recognises cargo bearing a classical NLS (cNLS, see Section 5.1) via importin-α, whereas importin-β directly interacts with components of the NPC to mediate nuclear translocation. Upon import of the importin-α/β-cargo complex, guanosine triphosphate (GTP)-bound Ran (RanGTP) binds to importin-β (Figure 1A). This causes the release of the cargo into the nucleus, and initiates the recycling of importin-α and importin-β-RanGTP back to the cytoplasm, where importin-β is released upon RanGTP hydrolysis [16] (Figure 1A).

### 2.2. Classical Nuclear Export

Exportin-1 is the primary mammalian nuclear export receptor and binds RanGTP in the nucleus to enhance its affinity for NES-bearing cargo [17,18] (see Section 5.2). Upon capture of a cargo, the exportin-1-RanGTP-cargo complex is exported to the cytoplasm and the hydrolysis of RanGTP to RanGDP releases the cargo in the cytoplasm [19]. RanGDP is then imported back into the nucleus [19,20,21,22] where it is converted back to RanGTP [23] (Figure 1B).

## 3. Intrinsic Disorder Is an Inherent Feature of the NPC

**Figure 2:**
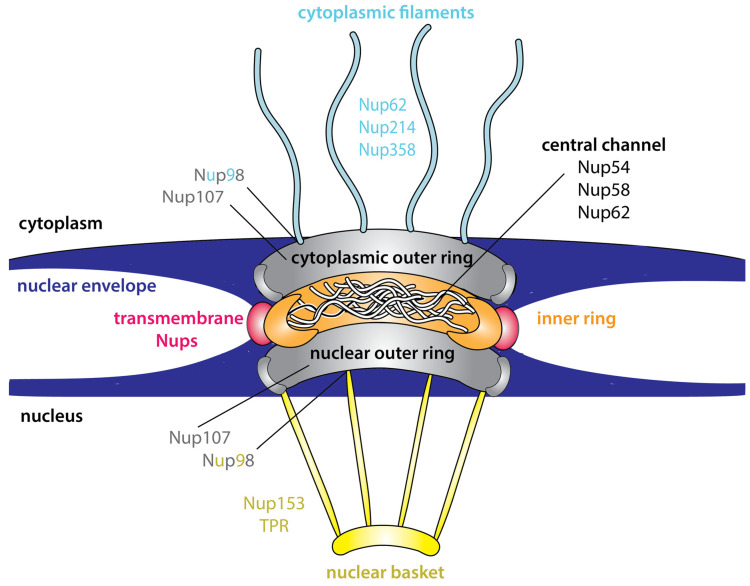
Cross-section of the nuclear pore complex (NPC). The NPC is comprised of two outer rings (grey) and an inner ring which forms the central channel of the pore. The inner ring is formed from layers of nucleoporins (Nups) that are collectively depicted in orange. The phenylalanine-glycine (FG)-Nups are predominantly anchored within the central channel and contain long extensions intermittently spaced with FG-repeats which have properties characteristic of intrinsically disordered proteins (IDPs). The FG-Nups form the permeability barrier of the NPC. The transmembrane Nups (red) anchor the NPC to the nuclear envelope. Nups and NPC proteins that are referred to in this review are shown. TPR = translocated promoter region.

The nuclear import/export of these nuclear transport receptor–cargo interactions occurs via the NPC. Each NPC is a megadalton-sized complex that is comprised of multiple copies of ~30 different proteins, known as nucleoporins (Nups). The Nups are generally grouped into three classes including the (1) transmembrane Nups that anchor the NPC to the nuclear envelope, (2) the structural Nups that form the two outer rings and inner ring of the NPC (through which the bi-directional transport of molecules between the nucleus and cytoplasm occurs), and (3) the phenylalanine-glycine (FG)-Nups that predominantly line the central channel of the NPC but are also localised to the filaments that project into the cytoplasm (cytoplasmic filaments, e.g., Nup214) and the nucleoplasm (nuclear basket e.g., Nup153) (Figure 2). The FG-Nups that are anchored into the central channel of the NPC do so via their coiled-coil domains, but contain long extensions, typically hundreds of amino acids in length [24]. The extensions contain long stretches of polar or charged residues interspersed with multiple FG-repeats [25]. The FG-repeats of the FG-Nups are intrinsically disordered such that overall the FG-Nups share characteristics with IDPs [26].

The FG-Nups collectively function as a soft barrier to passive diffusion; however, facilitated diffusion, or the active transport of molecules, can occur when molecules are accompanied by nuclear transport receptors. Specifically, the FG-repeats interact with multiple hydrophobic pockets on the surface of nuclear transport receptors [27,28,29,30]. These multiple low-affinity, transient interactions guide the passage of the nuclear import and export receptors, and their diverse cargo, through the NPC. However, how the FG-Nups form the permeability barrier of the NPC and also how, together with nuclear transport receptors, they allow the passage of larger cargoes remains controversial. The dynamic nature of the FG-Nups makes them difficult experimental targets, yet several models as to how this permeability barrier is formed have been proposed and here we briefly list three. In the “selective-phase” model it is proposed that the FG-repeats form hydrophobic interactions with one another, forming a selective sieve-like mesh/hydrogel, the size of which defines the soft molecular mass threshold of the NPC [21,31,32,33,34]. The interaction between nuclear transport receptors and the FG-repeats is proposed to disrupt the FG-repeat self-interactions, allowing the active passage of these larger complexes. In the “virtual-gate” and “polymer brush-like” models, the FG-repeats do not interact with one another and entropically exclude the transit of larger cargoes. The “virtual-gate” model proposes that the entropic barrier can be overcome by the binding of the FG-repeats to nuclear transport receptors [35]. Instead, the “polymer brush-like” model proposes that the FG-Nups are extended, but reversibly collapse towards their anchor point in the NPC when the FG-repeats bind nuclear transport receptors [36].

Regardless of the mechanism, it is clear that the FG-Nups provide a dense barrier within the NPC, and that the interaction between the FG-repeats and nuclear transport receptors is key to the timely passage of larger cargo through the NPC.

### Advantages of Intrinsic Disorder within the NPC

As proteins that behave as IDPs, the FG-Nups are ideally suited to mediate transport through the NPC. First, IDPs can accommodate a variety of binding partners [2,37], and can allow for simultaneous interactions [1,38]. These characteristics make them perfectly suited to allow multiple interactions with numerous nuclear transport receptors laden with or without diverse transport cargoes that may be simultaneously entering or exiting the nucleus. The ability of IDPs to adopt a range of conformations, at least in some models, is also likely key to allowing the above operations within the confined space of the NPC.

Second, IDPs can adopt multiple states in dynamic equilibrium and can exhibit varied behaviour under different environmental conditions. For example, under appropriate conditions, multivalent IDPs can undergo liquid-gel phase separation to from reversible hydrogels that are the basis of the “selective-phase” model of nuclear transport. Additionally, IDPs can also transition between extended-disordered and collapsed-disordered states [39], as per the “polymer brush-like” model.

Finally, nuclear transport is a selective, but fast process that occurs in milliseconds, and it is estimated that around 1000 molecules are transferred through a single NPC every second [21,40,41]. Passage across the NPC requires the binding of the hydrophobic patches on nuclear transport receptors with the FG-repeats, and their unbinding, in what is essentially a rapid exchange of transient interactions. NMR studies and molecular dynamics (MD) simulations show this rapid exchange is enabled through multiple low-affinity interactions, through which the FG-Nups remain inherently disordered [42,43], a feature that is ideally suited for rapid exchange [44].

## 4. Flexibility and Dynamics Are Inherent Features of Nuclear Transport Receptors

### 4.1. Importin α/β

Studies over the past two decades have highlighted that flexibility and dynamics are inherent features of nuclear transport receptors, including importin α/β and exportin-1. Importin-α is a ~60 kDa protein that is comprised of a short N-terminal importin-β binding (IBB) domain that is connected by a flexible linker to a larger C-terminal domain of 10 stacked helical armadillo (ARM) repeats. The ARM repeats, which form a super-helical solenoid structure, contain the major and minor NLS-binding sites of importin-α. The IBB domain, which is a stretch of around 40 predominantly basic amino acids that includes an NLS mimic, exists in an autoinhibitory conformation that is released upon binding to importin-β. The importin α/β heterodimer may then bind the NLS-containing cargo via importin-α, whereas importin-β interacts with components of the NPC. The importin α/β-cargo complex is then guided through the NPC to the nucleus. Once the importin α/β-cargo complex is imported into the nucleus, RanGTP binds to importin-β. This causes a region of importin-α to bind to the major NLS-binding site to competitively release the cargo. Thus, the flexible IBB domain is key to importin α/β binding, cargo uptake, nuclear transport and release. Accordingly, the X-ray structures of mouse and yeast importin-α revealed that the IBB domain is mostly disordered when not bound to importin-β [45,46]. MD simulations have also shown that while the IBB domain is in its α-helical form when bound to importin-β, the structure collapses in its unbound state [47].

Like importin-α, importin-β forms a super-helical solenoid structure, but has 19 HEAT repeats, each comprising two antiparallel helices connected by a turn [46]. Importin-β is considered to be more flexible than importin-α, displaying a diversity of conformations in its unbound and bound states, that range from compact to a more elongated configuration. Interestingly, this range of conformations has been observed in both static- and solution-based structural studies [46,48,49,50,51]. MD simulations further confirmed that importin-β sampled extreme and rapid conformations, including a more extended S-shaped conformation, due to flexibility in a number of the HEAT repeats [47,52]. Interestingly, when a RanGTP-bound form of importin-β was simulated, the degree of flexibility was significantly reduced [47]. These MD simulations also indicated that the free structure of importin-β displayed a degree of structural disorder, regardless of its well-defined secondary structure. The importin-β structure is only attenuated upon the binding of other proteins, and likely enables importin-β to bind cargoes of different size and shape. How this conformational flexibility dictates the function of nuclear transport has also been explored. Interestingly, decreasing importin-β flexibility via cross-linking resulted in hindered nuclear transport [53]. As mentioned previously, importin-β binds the IBB domain of importin-α, but it can also bind to other adaptors. In the absence of an adaptor importin-β can independently bind unrelated cargoes. Further to this, importin-β binds to the FG-Nups within the NPC and also to RanGTP within the nucleus, leading to the dissociation of the complex. It is therefore clear that the inherent flexibility of importin-β allows it to adjust to binding to different partners.

### 4.2. Exportin-1

**Figure 3:**
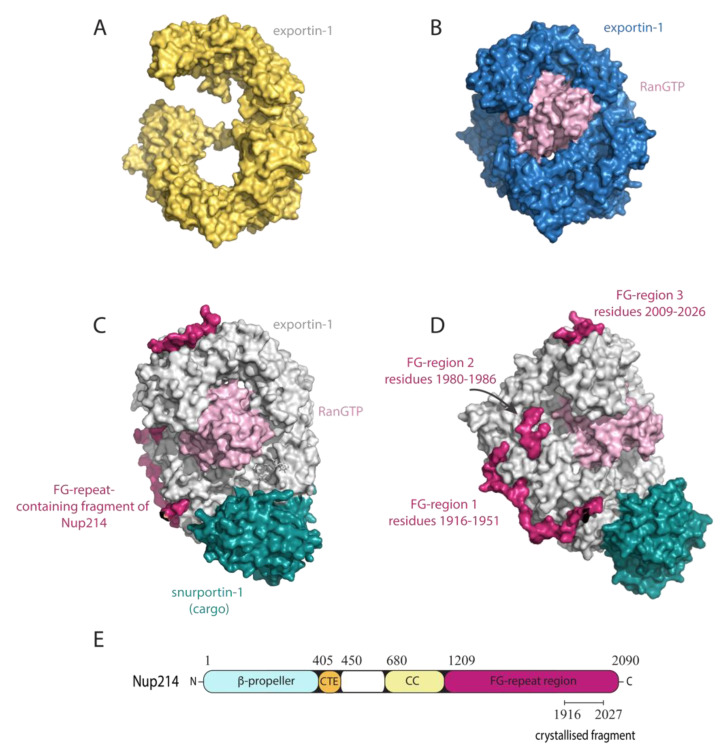
The nuclear export receptor exportin-1 adopts an extended or compact conformation and can bind to multiple partners. (**A**–**C**) Surface representations of exportin-1 depicted in the same orientation. (**A**) The extended conformation of unbound exportin-1 from fungus. Figure generated from Protein Data Bank (PDB) ID 4FGV [54]. (**B**) The compact ring-like conformation of mouse exportin-1 (blue) bound to RanGTP (pink). Figure generated from Protein Data Bank (PDB) ID 3NC1 [58]. (**C**,**D**) The human exportin-1-RanGTP-snurportin-1-Nup214 complex, where D is a rotation of C along the *Y*-axis. RanGTP (pink) interacts with the inner surface of exportin-1 (grey). The nuclear export signal (NES) of the transport cargo, in this case snurportin-1 (teal), interacts with the NES binding groove on the outer surface of exportin-1. The receptor-cargo complex is transported through the nuclear pore complex (NPC) via its interactions with FG-Nups, such as Nup214 (crimson), of which a fragment (residues 1916-2026) containing three FG-regions (FG-region 1-3) is shown. Each of the three FG-regions contain multiple FG-repeats and serve as anchor points for binding hydrophobic surface pockets of exportin-1. Residues connecting the FG-repeat regions were not always clearly defined in the electron density. Although the Nup214 fragment was crystallised with an N-terminal maltose binding protein (MBP) fusion tag, MBP has been removed from the figure for clarity. Figure generated from PDB ID 5DIS [27]. (**E**) Schematic representation of the domain architecture of Nup214, with the crystallised fragment that is shown in C,D depicted. CTE = C-terminal extension of the seven-bladed β-propeller; CC = coiled-coil.

Like importin-β, exportin-1 is comprised of 21 HEAT repeats and adopts an overall super-helical conformation (Figure 3A,B). The inner surface of exportin-1 interacts with RanGTP (Figure 3B), whereas the convex outer surface of exportin-1 interacts with the FG-Nups and harbours the NES-binding cleft to which cargo bind (Figure 3B–E). In both a crystalline and non-crystalline state, exportin-1 that is free of RanGTP and cargo can adopt both an extended conformation with high structural flexibility (Figure 3A) or a more rigid and compact ring-like conformation in which the N- and C-termini interact [54,55]. The more compact ring-like conformation is also observed when exportin-1 is bound to RanGTP in either the presence or absence of cargo (Figure 3B,C). Before it can bind cargo exportin-1 must first be bound by RanGTP. The cooperative nature of this binding is attributed to an acidic loop on exportin-1 that connects the two bound proteins, and motion in the C-terminal acidic tail that regulates the state of the NES binding cleft and the affinity of exportin-1 for its cargo [54,56,57].

## 5. Nuclear Transport Signals on Cargo Are Frequently Disordered

Experimentally verified importin α/β and exportin-1 cargoes have informed their consensus NLS or NES motifs, respectively, and whilst the consensus sequences vary, intrinsic disorder can be a common feature of each.

### 5.1. Nuclear Localisation Signals (NLSs)

Classical NLSs that are recognised by importin-α are either monopartite or bipartite. Monopartite NLSs contain one stretch of basic amino acids, whereas bipartite NLSs contain a second stretch of basic amino acids 10–12 residues downstream of the first stretch [59]. The consensus monopartite NLS sequence is defined as K-(K/R)-X-(K/R), where X can modulate NLS function and is typically enriched for positively charged residues [60,61,62,63]. Importin-α possesses two distinct NLS binding sites that are described as minor and major. The major NLS binding site binds to the classical monopartite NLS, whereas both the minor and major sites are required for binding to bipartite NLS [64,65]. Crystal structures have shown that classical NLS peptides bind to importin-α in an extended conformation. This suggests that conformational flexibility ensures favourable contacts are made with the binding site [61]. Intriguingly, classical NLSs are also often found in intrinsically disordered regions of cargo proteins [66,67,68]. One study that extracted NLS data from crystallographic structures showed that the mean B-factor per single amino acid of the NLS was significantly higher than the mean of the rest of the protein [69], confirming that classical NLSs often occur in regions of high mobility. However, it is important to note that ~20% of nuclear-localised importin α/β cargoes do not have a motif that matches the classical NLS [12]. Notably, an alternative motif, defined as K-R-X-H-X-K (where X is any amino acid), is also both rich in basic amino acids and predicted to be localised to IDRs [12].

### 5.2. Nuclear Export Signals (NESs)

The consensus NES is usually between 9-15 residues long and is defined as ϕ1-X_2,3_-ϕ2-X_2,3_-ϕ3-X-ϕ4, where ϕn represents a Leu, Val, Ile, Phe or Met residue and X represents any amino acid [70]. As per NLS binding to importin-α, exportin-1 binds to the NES motif of its cargo in an extended helix conformation [71], but can also adopt more diverse conformations [72]. The NES of the cargo binds within a groove of exportin-1 that contains multiple hydrophobic pockets. These pockets are proposed to allow for a variety of NES motifs to bind [71].

Secondary structural analysis has been conducted on known NES sequences [70,73], either from entries within the Protein Data Bank (PDB) or by using secondary structure prediction programs such as PSIPRED (Predict Secondary Structure). In a study by Xu et al., experimentally validated NES sequences were shown to have a strong bias towards helix-loop and loop conformations and a strong bias against β-strand conformations [73]. In the same study, the inherent disorder of NES motifs was also explored using the disorder prediction program DISOPRED2 [74]. Interestingly, the disorder propensity calculations predicted higher disorder scores for the experimentally proven NES sequences in comparison to the known false positives [73], supporting the thesis that protein disorder and solvent accessibility can be used to supplement the consensus motif as a tool for more accurate NES prediction [75,76].

### 5.3. Advantages of Disordered NLS/NES Motifs

There are many potential reasons as to why NLS and NES sequences are found in IDRs of cargo proteins. The intrinsic disorder may permit the solvent accessibility of the signal sequence, allowing the exposure of the key amino acids within the motif that are key to binding the nuclear transport receptor. In this regard it may also be key to allowing varied NLS/NES sequences, that still conform to the consensus motifs, to bind the structurally invariant NLS/NES binding site, and perhaps allow them to bind to the nuclear transport receptor in an optimal configuration. Finally, this flexibility may facilitate cargoes of different shapes and sizes to translocate through the NPC which has an upper functional diameter. While these possibilities make sense in the context of what is known about IDRs, they need to be further explored in the context of nuclear transport.

As a side note, 71% of 185 human nuclear proteins curated in the Nuclear Protein Database [77] are predicted to have ≥30% disorder [78], suggesting intrinsic disorder is abundant in proteins that localise to the nucleus (e.g., transcription factors [79]). Therefore, in this context intrinsic disorder appears to be a favourable feature.

## 6. Intrinsic Disorder Can Regulate the Nuclear Transport of Cargo

Given IDRs are hubs for protein-protein interactions and post-translational modifications, these factors, which can be sensitive to stimuli, frequently play a role in masking/unmasking NLS/NES motifs. The effect is to modulate the accessibility of cargo proteins to nuclear transport receptors, and in doing so regulate their cellular localisation. Examples of host cargo proteins whose NLS/NES motifs undergo masking/unmasking are described below.

### 6.1. The Integrase Interactor 1 (INI1) Protein

The integrase interactor 1 (INI1) protein, which is a subunit of the SWI/SNF (SWItch/Sucrose Non-Fermentable) chromatin remodelling complex, was reported to have a NES spanning residues 266-276 within the repeat 2 (Rpt2) domain. It was shown that in the steady state, the NES of INI1 is masked by its C-terminal arginine rich sequence (residues 363-378) [80]. It was postulated that the NES may be unmasked following a stimulus that either prevents a binding partner from blocking that region or causes a conformational change in the protein. We used the online software tool Predictors of Natural Disordered Regions (PONDR^®^) VL-XT [81], which hypothesises that the disordered structure characteristics of sequences depend on their location in the sequence [82], to predict disordered regions in INI1 (data not shown). Interestingly, PONDR^®^ VL-XT predicted that INI1 protein residues 356-381, which encompass the C-terminal NES masking sequence, are intrinsically disordered, suggesting that the conformational plasticity of these residues is involved in masking/unmasking events. Interestingly, INI1 is an oligomer and residues involved in oligomerisation overlap with those of the NES. Moreover, a double mutant (I264T/I268T) containing a key substitution in the NES (I268T) was both monomeric and strongly cytoplasmic, suggesting that multimerisation of INI1 masks the NES, causing the retention of INI1 in the nucleus [83].

### 6.2. Inhibitors of κBs

The nuclear factor κB (NF-κB) family of transcription factors require nuclear access to regulate the expression of genes in key cellular processes [84]. Inhibitors of κBs (IκBs), such as IκBα, block this nuclear localisation, rendering the NF-κB transcription factors inactive. Latimer et al. [85] showed that IκBα retains the p50 and cRel members of the NF-κB family in the cytoplasm, either by directly or indirectly masking one or both NLSs of the p50 and cRel homodimers. In particular, this masking was attributed to the N-terminal 61 residues of IκBα, which our PONDR^®^ VL-XT analysis predicted to be almost entirely disordered (58 of 61 residues; data not shown). Accordingly, the N-terminus was absent in the structure of IκBα in complex with the NF-κB heterodimer (p50/p65) [86]. This structure also emphasised the proximity of the stem of this flexible domain to the NLS of p50, where it appears conveniently positioned for its direct or indirect role in masking one NLS. Indeed, signalling induced phosphorylation of residues within the N-terminal disordered region of IκBα induces its ubiquitin-dependent degradation, releasing the transcription factors for nuclear entry. Cumulatively, the data strongly suggests that the malleability, and tuneability, of this N-terminal region plays a crucial role in regulating the masking/unmasking of the NLS.

Interestingly, IκBα also harbours an NLS within its disordered N-terminal region (residues 45-54), and the NLSs of the NF-κB family members p50, p52 and p65 [86,87,88,89] are also disordered, adding further complexity and dynamics to the system. Intrinsic disorder is generally recognised to be fundamental to transcription factors [79,90].

## 7. Intrinsic Disorder in Viral Proteins

Viruses are infectious agents that contain genetic material, DNA or RNA, enclosed by a protein capsid. For enveloped viruses, the capsid is surrounded by a lipid bilayer envelope that is derived from the host cell membrane. Viruses can only replicate by infecting their host, and given their viral genome is compact, they do so with a limited suite of viral proteins. For this reason, viruses interact with many host proteins during infection and depend on the host cellular machinery for their replication. Although viruses encode a limited number of viral proteins, this limitation can be mitigated by the fact that many viral proteins bear IDRs. In fact, in the manually curated Database of Protein Disorder (DisProt) [91] viral proteins constitute over 10% of the total entries across all organisms. The plasticity endowed by viral protein disorder has been correlated with virulence [92,93,94,95,96], potentially by hindering recognition by components of the immune system, but also allowing viral proteins to interact with multiple host factors, thereby maximising their function.

## 8. Viral Subversion of Host Nuclear Transport Machinery

Many viruses exploit nucleocytoplasmic shuttling pathways to access the nucleus of their host [97]. Viruses may require nuclear access to deliver their genome to the nucleus, and/or to subvert host cellular processes, such as the host immune response, to support their replication. For general viral subversion mechanisms of the nuclear transport machinery, the reader is referred to several excellent reviews [98,99]. Here however, our focus is to describe viral subversion mechanisms of the host nuclear transport machinery in which intrinsic disorder is a feature. 

### 8.1. FG-Nups Are Targeted by Viruses

During infection many viruses target multiple FG-Nups so as to alter the composition and function of the NPC, ultimately facilitating nuclear transport and infection. Below we list some prominent examples.

#### 8.1.1. Adenovirus Types 2 and 5

Adenovirus, which is a double stranded DNA virus, docks onto the cytoplasmic side of the NPC, and releases its viral genome from the capsid into the nucleus. More specifically, the adenovirus capsid protein, hexon, binds to the NPC via an IDR of Nup214, an FG repeat-containing Nup that is localised to the base of the cytoplasmic filament (Figure 2) [100,101]. The intact viral capsid protein, IX, then recruits kinesin-1 which binds another FG-Nup, Nup358 (Figure 2). This promotes adenovirus capsid uncoating, disrupts adenovirus capsids that are docked at the NPC, and releases multiple Nups from the nuclear envelope. The latter serves to increase the permeability of the nuclear envelope and allows viral genome access to the nucleus [102]. This process is aided by the adenovirus capsid protein VII, which strongly associates with adenovirus DNA and permits nuclear import by binding to numerous nuclear import receptors, including importin α/β, via its NLS-containing domains [103].

#### 8.1.2. Dengue and Zika Virus

Dengue virus (DENV) and Zika virus (ZIKV) are both flaviviruses and single-stranded RNA viruses that replicate in the host cell cytoplasm. Despite this, during infection many of their viral proteins enter the nucleus and many host proteins are mislocalised to aid viral replication or to inhibit innate immunity. These viruses alter the integrity of the NPC to achieve this outcome. The ZIKV and DENV serine-protease complex NS23B is responsible for degrading and altering the distribution of multiple FG-Nups following DENV and ZIKV infection. While both viruses target Nup98 and Nup153, an inner ring Nup and nuclear basket Nup, respectively (Figure 2), the degradation of the translocated promoter region (TPR) protein (scaffolding element of the NPC) and the channel FG-Nup Nup62 (Figure 2) were specific to ZIKV and DENV, respectively [104]. Although the degraded Nups are known to play roles in nuclear protein transport and mRNA export, further studies are required to determine how this strategy favours DENV and ZIKV replication.

#### 8.1.3. Poliovirus and Rhinovirus

Poliovirus and rhinovirus belong to the *Picornaviridae* family and are single stranded RNA viruses that replicate in the cytoplasm of the host cell. As per DENV and ZIKV, poliovirus and rhinovirus also cleave multiple Nups, but do so via their 3C and 2A cysteine proteases. Rhinovirus protease 2A cleaves Nup62, Nup98 and Nup153 [105,106,107,108], whereas protease 3C cleaves Nup153, Nup214 and Nup358 [109], and both Nup98 and Nup153 are also degraded by poliovirus [110,111,112]; notably, all of these Nups are FG-Nups (Figure 2). Characterisation of the rhinovirus 2A protease Nup98 cleavage sites revealed that proteolysis removes the N-terminal FG-repeat region from the NPC, while leaving the C-terminus anchored to the NPC [113]. These viruses therefore are able to alter the composition of the NPC to influence its permeability and nuclear transport in infected cells [110,111,114]. However, it should be noted that these viral proteases also cleave host proteins beyond the aforementioned Nups, also to favour viral replication (e.g., [3]).

#### 8.1.4. Encephalomyocarditis Virus (EMCV)

Encephalomyocarditis virus (EMCV), which also belongs to the *Picornaviridae* family, infects a broad range of non-human hosts; however, human infections associated with clinical manifestations have been reported [115,116]. Although EMCV is a picornavirus, as per poliovirus and rhinovirus, it does not encode the 2A protease and is incapable of cleaving NPC Nups during infection. Rather, EMCV regulates nuclear trafficking via its variable-length Leader (L) protein which lacks enzymatic activity. In this capacity, L, or more specifically its N-terminal zinc-finger motif, is key to multiple approaches to disrupt nucleocytoplasmic trafficking, including binding directly to Ran [117], and altering patterns of FG-Nup phosphorylation (e.g., Nup62, Nup98, Nup153 and Nup214 [118,119,120]) (Figure 2). Cumulatively, these strategies modulate Ran-dependent nucleocytoplasmic shuttling to relocate proteins in favour of viral replication and interferon (IFN) suppression [117,121], and alter the kinetics of active nuclear transport [118,119,120,122,123]. MD simulations suggest that phosphorylation of isolated FG-Nup segments alters the kinetics of transport by reducing their intramolecular cohesion and leading to a more extended FG-Nup conformation. Further to this, phosphorylated NPCs (where all the serine and threonine residues of the FG-Nups are phosphorylated) had reduced protein density compared to wild-type NPCs [124]. It should be noted; however, that the portion of phosphorylated residues at any one time inside the NPC in vivo is not known.

#### 8.1.5. Hepatitis C Virus (HCV)

Although HCV is an RNA virus that replicates in the cytoplasm, it utilises both Nups and nuclear transport receptors to support infection. HCV infection leads to the recruitment and rearrangement of cytoplasmic host cell membranes into viral replication and assembly complexes. Intriguingly, NLS-bearing proteins and Nups are recruited to these HCV assembly sites during infection [125]. This change in Nup localisation was also associated with upregulated Nup mRNA and protein levels including, but not limited to, Nup153 and Nup214 (both FG-Nups) [125]. Further to this, the HCV core and NS5A proteins were found to interact with Nup107 (Figure 2) and Nup153 [125] and were amongst the HCV proteins that were reported to interact with nuclear transport receptors such as exportin-1 [125,126]. Therefore, nuclear transport proteins function in HCV replication, but the precise details require further clarification.

### 8.2. Intrinsic Disorder Can Modulate Viral Cargo NLS/NES Accessibility

A common mechanism for viral proteins to enter or exit the nucleus of host cells is to mimic host NLS/NES motifs, respectively, to hijack host nuclear transport receptors. Over the past ten years, there has been a surge in the experimental validation of such motifs within viral proteins. For example, viral proteins, such as human immunodeficiency virus type 1 (HIV-1) Rev [127] and influenza virus nucleoprotein (NP) and polymerase basic protein 2 (PB2) [128,129] mimic the host NLS and enter the nucleus via importin α/β. Likewise, viral proteins such as Hendra virus V [130], HIV-1 Rev [127], respiratory syncytial virus matrix (M) protein [131] and influenza virus NS2 (nuclear export protein) [132], amongst many others, mimic host NES sequences to bind exportin-1 for their nuclear export. As per human proteins, many of these viral NLS and NES motifs are reported to occur within disordered regions. However, we have further extended the list by using PONDR^®^ VL-XT to predict disorder within experimentally validated viral nuclear import and export signals (Table 1). Our findings suggest that more viral NLS/NES motifs are potentially disordered than currently appreciated, a feature which should be explored in the context of nuclear transport.

As per NLS/NES motifs in human proteins, viral proteins are also subjected to mechanisms that regulate their nuclear transport. This includes the modulation of NLS/NES accessibility by intra- and inter-molecular interactions and post-translational modifications. Intrinsic disorder, which is abundant in viral proteins, plays a key role in this modulation.

#### 8.2.1. Human Immunodeficiency Virus Type 1 (HIV-1) Rev

Rev is a HIV-1 regulatory protein that is essential to the HIV-1 life cycle. Following its translation in the cytoplasm, Rev is imported into the nucleus where it binds to Rev responsive elements (RREs) on intron-containing HIV-1 mRNA and exports them to the cytoplasm for translation. To perform this function, Rev requires access to both the nuclear and cytoplasmic compartments of the cell, and this shuttling must be tightly regulated to ensure it is in the right compartment at the right time. The structural/biophysical/cellular analysis of Rev has provided insights into how this might occur.

RNA-free Rev crystal structures show that the N-terminal domain consists of two helical oligomerisation domains (residues 9-26 and 51-65) connected by a flexible linker [139,140]. The second helical domain is flanked by an α-helical arginine rich motif (ARM) which binds to the RRE-containing RNA and also functions as an NLS (residues 34-50) that directly interacts with importin-β [127,141,142,143]. In addition to its α-helical conformation, the 17 amino acid ARM/NLS peptide has also been observed in an extended conformation, with the conformation dictated by the RNA to which it was bound [144]. The conformational variability of the ligand-free ARM/NLS peptide is also supported by Casu et al. [145]. Further to this, the C-terminal domain of Rev (residues 66-116), that harbours the NES (residues 74-83) that is recognised by exportin-1, is disordered [139,146,147].

Rev’s intra- and intermolecular protein interactions, RRE-binding status and the conformational malleability provided by its flexible linkers and intrinsically disordered regions are proposed to dictate NLS/NES masking and unmasking events. Rev self-association within the cytoplasm is proposed to transiently mask the NES, thereby promoting its NLS-mediated trafficking to the nucleus [148] where it can access the RRE on intron-containing HIV-1 mRNA. Within the nucleus, it is proposed that the NLS of Rev is masked upon binding the RRE [149]. For a functional nuclear export complex, Rev must form higher order oligomers along the RRE [150], and the NES within Rev’s disordered C-terminal domain must be exposed for its interaction with exportin-1-RanGTP. Given the C-terminal disordered domain of Rev regulates its stability and oligomerisation [151], the assembly of higher order Rev oligomers on the RRE may facilitate the exposure of the NES [139,152]. Regardless of the mechanism, it is clear that the intrinsically disordered elements within Rev are paramount to its malleability, stability and modulation of its protein-protein and protein-RNA interactions, enabling its functional versatility.

#### 8.2.2. Hepatitis C Virus (HCV) Core

Although HCV replicates in the cytoplasm of infected cells, many HCV viral proteins, including the HCV core, shuttle between the cytoplasm and the nucleus to promote viral replication [125,126,153,154]. To identify HCV proteins carrying nuclear transport signals, one study incubated a HCV peptide array with/without cell lysates and probed the array with antibodies that recognise components of the nuclear transport machinery. In doing so they identified three NLSs within the HCV core protein that bind to both importin-α5 and importin-β3 [126]. Our PONDR^®^ VL-XT analysis predicts that all three NLSs (NLS1-3) within the HCV core protein are fully disordered (Table 1).

Biophysical/NMR analysis revealed that the mature core protein (residues 1-177) contains stable secondary structure and exists as an oligomer [155,156]. However, an N-terminal truncation of the core (residues 1-82) was intrinsically disordered [157], and a C-terminal truncation (residues 1-124) was both intrinsically disordered and monomeric [155]. These findings suggest that the C-terminal tail is important for oligomerisation, and that C-terminal intramolecular interactions induce structure within the HCV core. We speculate that NLS masking, induced by ligand-binding and/or oligomerisation, may play a role in regulating the nuclear import of the HCV core protein. Although the original study that identified NLS1-3 reported that their recombinant full-length core protein (residues 1-191) bound importin-α5 and importin-β3, neither the oligomeric state of this precursor core protein, nor whether it was proteolytically cleaved, was reported [126].

#### 8.2.3. Hendra Virus P/V/W

We previously showed that the Hendra virus V protein shuttles between the nucleus and the cytoplasm via interaction with importin α/β and exportin-1, respectively [130]. We experimentally validated a NES (residues 174-192) within the large disordered N-terminal domain of the Hendra virus V protein (residues 1-405) that is also shared with the phosphoprotein (P) and W protein of Hendra virus. Although P/V/W share their disordered N-terminal domains, they have distinct, structurally ordered C-terminal domains. Yet, despite carrying a common NES, only P and V are localised to the cytoplasm at steady state. The nuclear localisation of the W protein at steady state suggests its localisation is predominantly determined by its NLS at its C-terminus, and that the N-terminal NES it shares with P/V may be non-functional, or is perhaps masked as can occur with HIV-1 Rev. Likewise, the V and W proteins of the closely related Nipah virus also differentially localise to the cytoplasm and nucleus, respectively [158,159], suggesting a regulatory mechanism common to their Hendra virus counterparts. These viral proteins are critical to the pathogenesis of these henipaviruses and serve to limit or prevent host type I IFN induction from different cellular compartments.

#### 8.2.4. Simian Virus 40 (SV-40) Large T-Antigen

Phosphorylation sites in close proximity to NLS/NES motifs can often up- or down-regulate the nuclear import/export of cargoes. For example, casein kinase 2 (CK2) phosphorylates residues S111/S112 of the Simian virus 40 (SV40) large T-antigen (T-ag), which are immediately upstream of an NLS (residues 126-132). This phosphorylation event accelerates the importin α/β-mediated import of T-ag, although a crystal structure confirmed there was no direct interaction between the S112 phosphate moiety and importin-α [62,64,160,161]. Another structural study compared the phosphorylated and non-phosphorylated T-Ag NLS peptides. They showed that unlike the phosphorylated peptide, which displayed continuous electron density from residues 119-133, there was no discernible electron density for the non-phosphorylated peptide between residues 119-122, suggesting this region is disordered. Accordingly, our PONDR^®^ VL-XT analysis predicts that SV40 T-ag residues 104-139, spanning both the NLS and the upstream phosphorylation sites, are disordered (data not shown). These data suggest that the phosphorylation of residues upstream of the T-ag NLS may prompt residues 119-122 to undergo a disorder–order transition that deems the NLS more recognisable by importin-α [62,64].

## 9. Therapeutic Targeting of Nuclear Transport to Limit Viral Infection

Given many viruses are dependent on accessing the host nucleus to replicate their genome, or to suppress the host antiviral response, nuclear transport inhibitors that mislocalise host or viral proteins key to the viral life cycle, are emerging as promising antiviral therapeutics.

### 9.1. Nuclear Import Inhibitors

Ivermectin is a small molecule that impairs viral replication by various means. Two independent in vitro studies concur that ivermectin binds to importin-α and in doing so induces structural changes that prevent the binding of NLS-bearing cargo [162,163]. However, only one of these studies shows that these structural changes caused the dissociation of the importin α/β heterodimer. Consistent with preventing the binding of NLS-bearing cargo, ivermectin inhibits the nuclear import of host [164] and viral proteins [163,165,166,167]. Therefore, ivermectin may disable viruses that are reliant on importin α/β during infection. However, ivermectin has also been shown to bind to the non-structural protein 3 (NS3) of two flaviviruses, West Nile virus (WNV) and yellow fever virus (YFV), and in doing so inhibit their double stranded RNA unwinding activity [168]. Accordingly, ivermectin inhibits the replication of WNV and YFV in vitro [162,168]. Notably however, ivermectin’s antiviral activity is broader than just these two flaviviruses and includes other human viruses. These viruses include, but not are not limited to, Hendra virus [130], Venezuelan equine encephalitis virus [169], ZIKV [162,169,170,171], DENV [168,172,173], influenza [174], HIV-1 [171] and even SARS-CoV-2 [175], the causative agent of COVID-19. Encouragingly, in mice, ivermectin has also been shown to relieve pseudorabies virus infection by inhibiting a subunit of its DNA polymerase from entering the nucleus [166]. However, in contrast, and despite the demonstrated anti-ZIKV activity of ivermectin in vitro, ivermectin treatment failed to protect mice from lethal ZIKV infection [176]. Collectively these findings suggest that ivermectin has antiviral properties in vitro, but it is important to determine if these results are corroborated in small animal models and also in a clinical context. Notably, ivermectin, which is an FDA-approved anti-parasitic drug, is the subject of numerous clinical trials in human patients to determine if it can safely reduce the SARS-CoV-2 viral load on its own, or in combination with other agents (e.g., NCT04381884, NCT04425707).

Likewise, karyostatin 1A [177] and importazole [178] are small molecule nuclear import inhibitors. Specifically, these inhibitors prevent, or alter importin-β’s interaction with RanGTP, respectively, and may also have therapeutic application.

### 9.2. Nuclear Export Inhibitors

Nuclear export can also be targeted by small molecules. Leptomycin B (LMB), the prototypical exportin-1 inhibitor, binds irreversibly to cysteine 528 within the NES binding groove [179]. In doing so, LMB blocks the binding of all NES-bearing cargo to exportin-1 and prevents their nuclear export, causing their nuclear accumulation. In cells, LMB inhibits the nuclear export of the HIV-1 Rev protein, which is essential for the export of unspliced and incompletely spliced HIV-1 mRNA, and limits HIV-1 replication [180]. LMB treatment of influenza virus infected cells also causes the nuclear retention of NP and blocks the export of ribonucleoproteins therein preventing their translation in the cytoplasm and limiting influenza virus replication [181]. We also reported that LMB limits Hendra virus infection, although the critical human or viral host protein/s that have been mislocalised in this instance remain unknown [130]. Although LMB demonstrated antiviral properties in vitro, clinical trials were abandoned due to its toxicity [182]. LMB has since been superseded by agents, such as selinexor, that reversibly bind cysteine 528 of exportin-1. In 2020 selinexor was granted FDA-approval for treating patients with refractory diffuse large B-cell lymphoma (DLBCL), and is undergoing clinical trials in patients suffering from severe SARS-CoV-2 infection (e.g., NCT04349098, NCT04355676). Likewise, second-generation compounds, such as eltanexor (KPT-8602), which also target exportin-1, but have superior properties to selinexor [183,184], show promise for cancer indications, and should also be tested for their antiviral properties.

### 9.3. Cargo-Specific Transport Inhibitors

Despite the demonstrated utility of nuclear import and export inhibitors in reducing viral replication in vitro, there are currently no cargo-specific nuclear export inhibitors. Such inhibitors will be critically important to maintain specificity and reduce the cytotoxic effects associated with globally inhibiting nuclear transport cargo. To develop cargo-specific inhibitors, extensive knowledge of the cargo-receptor interface is required. In this regard, our knowledge is most advanced for the classical NLS recognised by importin α/β and the NES recognised by exportin-1, and so there is perhaps the greatest potential to selectively disrupt NLS- or NES-bearing cargo interactions. However, as noted above, NLS and NES motifs often contain regions of disorder, and in lacking stable features, are considered challenging drug targets. Although previously considered “undruggable” targets, success stories demonstrating that small molecules can selectively bind to disordered protein targets are emerging [185,186,187,188]. Future progress in defining critical cargo–receptor interactions, and their importance to the life cycle of viruses, will fuel the development of cargo-specific nuclear transport inhibitors with highly specific activity, low toxicity and great promise for viral intervention.

## 10. Conclusions

The FG-Nups that reside within the NPCs that perforate the nuclear envelope provide a selective barrier that regulates the movement of molecules between the cytoplasm and the nucleus. Accordingly, nucleocytoplasmic transport is essential for many critical functions of the cell, and also to the replication strategy of viruses infecting their host. It is clear that inherent disorder/flexibility plays a role, at various levels, to the fundamental dynamics of nuclear transport. However, due to these very dynamics, understanding the consequence of protein flexibility and disorder to trafficking is considerably challenging. It is clear more approaches and methodologies are required to further clarify the role of inherent disorder in the operation, and host- and viral-mediated regulation, of the extraordinarily complex and vital nuclear transport machinery. Ultimately, an enhanced understanding is key to devising novel therapies to block the replication strategy of viruses.

## Figures and Tables

**Table 1:** PONDR^®^ VL-XT-predicted disorder of experimentally validated NLS/NES motifs.

Virus	Protein	NLS/NES	Sequence	Ref.
PYDV ^1^	M	NLS	213-KKTV**SDPLKNLLKR**-226	[133]
HIV-1 ^2^	Capsid	NLS	84-**HPVHAGPIAPGQMREPRGSDIA**-105	[134]
Measles	C	NLS	41-**PPARKRRQ**-48	[135]
HCV ^3^	Core	NLS1	6-**KPQRKTKRN**-14	[126]
HCV	Core	NLS2	38-**PRRGPR**-43	[126]
HCV	Core	NLS3	58-**PRGRRQPIPKARRS**-71	[126]
BDV ^4^	N	NLS	3-**PKRRLVDDT**-11	[136]
HTLV-1 ^5^	Rex	NES	81-**ALSAQLYSSLSLDSP**-95	[137]
HTLV-1	Rex	NLS	1-**MPKTRRRPRRSQRKRPPTP**-19	[138]

1: Potato yellow dwarf nucleorhabdovirus; 2: Human immunodeficiency virus type 1; 3: Hepatitis C; 4: Borna disease virus; 5: Human T-cell leukaemia virus type I. NLS = nuclear localisation signal; NES = nuclear export signal. Disordered residues depicted in bold.

## References

[1] Uversky V.N. (2002). Natively unfolded proteins: A point where biology waits for physics. Protein Sci..

[2] Uversky V.N. (2002). What does it mean to be natively unfolded?. Eur. J. Biochem..

[3] Croft S.N., Walker E.J., Ghildyal R. (2018). Human Rhinovirus 3C protease cleaves RIPK1, concurrent with caspase 8 activation. Sci. Rep..

[4] Dyson H.J., Wright P.E. (2005). Intrinsically unstructured proteins and their functions. Nat. Rev. Mol. Cell Biol..

[5] Wright P.E., Dyson H.J. (2009). Linking folding and binding. Curr. Opin. Struct. Biol..

[6] Shammas S.L., Crabtree M.D., Dahal L., Wicky B.I., Clarke J. (2016). Insights into Coupled Folding and Binding Mechanisms from Kinetic Studies. J. Biol. Chem..

[7] Ittisoponpisan S., Alhuzimi E., Sternberg M.J., David A. (2017). Landscape of Pleiotropic Proteins Causing Human Disease: Structural and System Biology Insights. Hum. Mutat..

[8] Iakoucheva L.M., Radivojac P., Brown C.J., O’Connor T.R., Sikes J.G., Obradovic Z., Dunker A.K. (2004). The importance of intrinsic disorder for protein phosphorylation. Nucleic Acids Res..

[9] Wright P.E., Dyson H.J. (2015). Intrinsically disordered proteins in cellular signalling and regulation. Nat. Rev. Mol. Cell. Biol..

[10] Timney B.L., Raveh B., Mironska R., Trivedi J.M., Kim S.J., Russel D., Wente S.R., Sali A., Rout M.P. (2016). Simple rules for passive diffusion through the nuclear pore complex. J. Cell Biol..

[11] Kimura F., Suzu S., Nakamura Y., Nakata Y., Yamada M., Kuwada N., Matsumura T., Yamashita T., Ikeda T., Sato K. (2003). Cloning and characterization of a novel RING-B-box-coiled-coil protein with apoptotic function. J. Biol. Chem..

[12] Tessier T.M., MacNeil K.M., Mymryk J.S. (2020). Piggybacking on Classical Import and Other Non-Classical Mechanisms of Nuclear Import Appear Highly Prevalent within the Human Proteome. Biology.

[13] Berneking L., Schnapp M., Rumm A., Trasak C., Ruckdeschel K., Alawi M., Grundhoff A., Kikhney A.G., Koch-Nolte F., Buck F. (2016). Immunosuppressive Yersinia Effector YopM Binds DEAD Box Helicase DDX3 to Control Ribosomal S6 Kinase in the Nucleus of Host Cells. PLoS Pathog..

[14] Kimura M., Imamoto N. (2014). Biological significance of the importin-beta family-dependent nucleocytoplasmic transport pathways. Traffic.

[15] Goldfarb D.S., Corbett A.H., Mason D.A., Harreman M.T., Adam S.A. (2004). Importin alpha: A multipurpose nuclear-transport receptor. Trends Cell Biol..

[16] Kutay U., Bischoff F.R., Kostka S., Kraft R., Gorlich D. (1997). Export of importin alpha from the nucleus is mediated by a specific nuclear transport factor. Cell.

[17] Lindsay M.E., Holaska J.M., Welch K., Paschal B.M., Macara I.G. (2001). Ran-binding protein 3 is a cofactor for Crm1-mediated nuclear protein export. J. Cell Biol..

[18] Fornerod M., Ohno M., Yoshida M., Mattaj I.W. (1997). CRM1 is an export receptor for leucine-rich nuclear export signals. Cell.

[19] Gorlich D., Pante N., Kutay U., Aebi U., Bischoff F.R. (1996). Identification of different roles for RanGDP and RanGTP in nuclear protein import. EMBO J..

[20] Smith A., Brownawell A., Macara I.G. (1998). Nuclear import of Ran is mediated by the transport factor NTF2. Curr. Biol..

[21] Ribbeck K., Gorlich D. (2001). Kinetic analysis of translocation through nuclear pore complexes. EMBO J..

[22] Moore M.S., Blobel G. (1994). Purification of a Ran-interacting protein that is required for protein import into the nucleus. Proc. Natl. Acad. Sci. USA.

[23] Bischoff F.R., Ponstingl H. (1991). Catalysis of guanine nucleotide exchange on Ran by the mitotic regulator RCC1. Nature.

[24] Fried H., Kutay U. (2003). Nucleocytoplasmic transport: Taking an inventory. Cell Mol. Life Sci..

[25] Rout M.P., Wente S.R. (1994). Pores for thought: Nuclear pore complex proteins. Trends Cell Biol..

[26] Denning D.P., Patel S.S., Uversky V., Fink A.L., Rexach M. (2003). Disorder in the nuclear pore complex: The FG repeat regions of nucleoporins are natively unfolded. Proc. Natl. Acad. Sci. USA.

[27] Port S.A., Monecke T., Dickmanns A., Spillner C., Hofele R., Urlaub H., Ficner R., Kehlenbach R.H. (2015). Structural and Functional Characterization of CRM1-Nup214 Interactions Reveals Multiple FG-Binding Sites Involved in Nuclear Export. Cell Rep..

[28] Koyama M., Shirai N., Matsuura Y. (2014). Structural insights into how Yrb2p accelerates the assembly of the Xpo1p nuclear export complex. Cell Rep..

[29] Koyama M., Hirano H., Shirai N., Matsuura Y. (2017). Crystal structure of the Xpo1p nuclear export complex bound to the SxFG/PxFG repeats of the nucleoporin Nup42p. Genes Cells.

[30] Bayliss R., Kent H.M., Corbett A.H., Stewart M. (2000). Crystallization and initial X-ray diffraction characterization of complexes of FxFG nucleoporin repeats with nuclear transport factors. J. Struct. Biol..

[31] Mohr D., Frey S., Fischer T., Guttler T., Gorlich D. (2009). Characterisation of the passive permeability barrier of nuclear pore complexes. EMBO J..

[32] Keminer O., Peters R. (1999). Permeability of single nuclear pores. Biophys. J..

[33] Frey S., Gorlich D. (2009). FG/FxFG as well as GLFG repeats form a selective permeability barrier with self-healing properties. EMBO J..

[34] Frey S., Gorlich D. (2007). A saturated FG-repeat hydrogel can reproduce the permeability properties of nuclear pore complexes. Cell.

[35] Rout M.P., Aitchison J.D., Magnasco M.O., Chait B.T. (2003). Virtual gating and nuclear transport: The hole picture. Trends Cell Biol..

[36] Lim R.Y., Fahrenkrog B., Koser J., Schwarz-Herion K., Deng J., Aebi U. (2007). Nanomechanical basis of selective gating by the nuclear pore complex. Science.

[37] Dunker A.K., Lawson J.D., Brown C.J., Williams R.M., Romero P., Oh J.S., Oldfield C.J., Campen A.M., Ratliff C.M., Hipps K.W. (2001). Intrinsically disordered protein. J. Mol. Graph. Model..

[38] Dyson H.J., Wright P.E. (2018). How Do Intrinsically Disordered Viral Proteins Hijack the Cell?. Biochemistry.

[39] Dunker A.K., Obradovic Z. (2001). The protein trinity--linking function and disorder. Nat. Biotechnol..

[40] Yang W., Gelles J., Musser S.M. (2004). Imaging of single-molecule translocation through nuclear pore complexes. Proc. Natl. Acad. Sci. USA.

[41] Kubitscheck U., Grunwald D., Hoekstra A., Rohleder D., Kues T., Siebrasse J.P., Peters R. (2005). Nuclear transport of single molecules: Dwell times at the nuclear pore complex. J. Cell Biol..

[42] Hough L.E., Dutta K., Sparks S., Temel D.B., Kamal A., Tetenbaum-Novatt J., Rout M.P., Cowburn D. (2015). The molecular mechanism of nuclear transport revealed by atomic-scale measurements. Elife.

[43] Raveh B., Karp J.M., Sparks S., Dutta K., Rout M.P., Sali A., Cowburn D. (2016). Slide-and-exchange mechanism for rapid and selective transport through the nuclear pore complex. Proc. Natl. Acad. Sci USA.

[44] Schneider R., Maurin D., Communie G., Kragelj J., Hansen D.F., Ruigrok R.W., Jensen M.R., Blackledge M. (2015). Visualizing the molecular recognition trajectory of an intrinsically disordered protein using multinuclear relaxation dispersion NMR. J. Am. Chem. Soc..

[45] Kobe B. (1999). Autoinhibition by an internal nuclear localization signal revealed by the crystal structure of mammalian importin alpha. Nat. Struct. Biol..

[46] Cingolani G., Petosa C., Weis K., Muller C.W. (1999). Structure of importin-beta bound to the IBB domain of importin-alpha. Nature.

[47] Zachariae U., Grubmuller H. (2008). Importin-beta: Structural and dynamic determinants of a molecular spring. Structure.

[48] Vetter I.R., Arndt A., Kutay U., Gorlich D., Wittinghofer A. (1999). Structural view of the Ran-Importin beta interaction at 2.3 A resolution. Cell.

[49] Liu S.M., Stewart M. (2005). Structural basis for the high-affinity binding of nucleoporin Nup1p to the Saccharomyces cerevisiae importin-beta homologue, Kap95p. J. Mol. Biol..

[50] Lee S.J., Imamoto N., Sakai H., Nakagawa A., Kose S., Koike M., Yamamoto M., Kumasaka T., Yoneda Y., Tsukihara T. (2000). The adoption of a twisted structure of importin-beta is essential for the protein-protein interaction required for nuclear transport. J. Mol. Biol..

[51] Bhardwaj A., Cingolani G. (2010). Conformational selection in the recognition of the snurportin importin beta binding domain by importin beta. Biochemistry.

[52] Forwood J.K., Lange A., Zachariae U., Marfori M., Preast C., Grubmuller H., Stewart M., Corbett A.H., Kobe B. (2010). Quantitative structural analysis of importin-beta flexibility: Paradigm for solenoid protein structures. Structure.

[53] Yoshimura S.H., Kumeta M., Takeyasu K. (2014). Structural mechanism of nuclear transport mediated by importin beta and flexible amphiphilic proteins. Structure.

[54] Monecke T., Haselbach D., Voss B., Russek A., Neumann P., Thomson E., Hurt E., Zachariae U., Stark H., Grubmuller H. (2013). Structural basis for cooperativity of CRM1 export complex formation. Proc. Natl. Acad. Sci. USA.

[55] Dolker N., Blanchet C.E., Voss B., Haselbach D., Kappel C., Monecke T., Svergun D.I., Stark H., Ficner R., Zachariae U. (2013). Structural determinants and mechanism of mammalian CRM1 allostery. Structure.

[56] Koyama M., Matsuura Y. (2010). An allosteric mechanism to displace nuclear export cargo from CRM1 and RanGTP by RanBP1. EMBO J..

[57] Fox A.M., Ciziene D., McLaughlin S.H., Stewart M. (2011). Electrostatic interactions involving the extreme C terminus of nuclear export factor CRM1 modulate its affinity for cargo. J. Biol. Chem..

[58] Guttler T., Madl T., Neumann P., Deichsel D., Corsini L., Monecke T., Ficner R., Sattler M., Gorlich D. (2010). NES consensus redefined by structures of PKI-type and Rev-type nuclear export signals bound to CRM1. Nat. Struct. Mol. Biol..

[59] Lange A., Mills R.E., Lange C.J., Stewart M., Devine S.E., Corbett A.H. (2007). Classical nuclear localization signals: Definition, function, and interaction with importin alpha. J. Biol. Chem..

[60] Smith K.M., Di Antonio V., Bellucci L., Thomas D.R., Caporuscio F., Ciccarese F., Ghassabian H., Wagstaff K.M., Forwood J.K., Jans D.A. (2018). Contribution of the residue at position 4 within classical nuclear localization signals to modulating interaction with importins and nuclear targeting. Biochim. Biophys. Acta Mol. Cell Res..

[61] Marfori M., Lonhienne T.G., Forwood J.K., Kobe B. (2012). Structural basis of high-affinity nuclear localization signal interactions with importin-alpha. Traffic.

[62] Fontes M.R., Teh T., Kobe B. (2000). Structural basis of recognition of monopartite and bipartite nuclear localization sequences by mammalian importin-alpha. J. Mol. Biol..

[63] Chelsky D., Ralph R., Jonak G. (1989). Sequence requirements for synthetic peptide-mediated translocation to the nucleus. Mol. Cell Biol..

[64] Fontes M.R., Teh T., Toth G., John A., Pavo I., Jans D.A., Kobe B. (2003). Role of flanking sequences and phosphorylation in the recognition of the simian-virus-40 large T-antigen nuclear localization sequences by importin-alpha. Biochem. J..

[65] Conti E., Uy M., Leighton L., Blobel G., Kuriyan J. (1998). Crystallographic analysis of the recognition of a nuclear localization signal by the nuclear import factor karyopherin alpha. Cell.

[66] Morita E.H., Shirakawa M., Hayashi F., Imagawa M., Kyogoku Y. (1995). Structure of the Oct-3 POU-homeodomain in solution, as determined by triple resonance heteronuclear multidimensional NMR spectroscopy. Protein Sci..

[67] Galea C.A., Wang Y., Sivakolundu S.G., Kriwacki R.W. (2008). Regulation of cell division by intrinsically unstructured proteins: Intrinsic flexibility, modularity, and signaling conduits. Biochemistry.

[68] Cervantes C.F., Bergqvist S., Kjaergaard M., Kroon G., Sue S.C., Dyson H.J., Komives E.A. (2011). The RelA nuclear localization signal folds upon binding to IkappaBalpha. J. Mol. Biol..

[69] Yamagishi R., Okuyama T., Oba S., Shimada J., Chaen S., Kaneko H. (2015). Comprehensive analysis of the dynamic structure of nuclear localization signals. Biochem. Biophys. Rep..

[70] la Cour T., Kiemer L., Molgaard A., Gupta R., Skriver K., Brunak S. (2004). Analysis and prediction of leucine-rich nuclear export signals. Protein Eng. Des. Sel..

[71] Dong X., Biswas A., Suel K.E., Jackson L.K., Martinez R., Gu H., Chook Y.M. (2009). Structural basis for leucine-rich nuclear export signal recognition by CRM1. Nature.

[72] Fung H.Y., Fu S.C., Chook Y.M. (2017). Nuclear export receptor CRM1 recognizes diverse conformations in nuclear export signals. Elife.

[73] Xu D., Farmer A., Collett G., Grishin N.V., Chook Y.M. (2012). Sequence and structural analyses of nuclear export signals in the NESdb database. Mol. Biol. Cell.

[74] Ward J.J., Sodhi J.S., McGuffin L.J., Buxton B.F., Jones D.T. (2004). Prediction and functional analysis of native disorder in proteins from the three kingdoms of life. J. Mol. Biol..

[75] Xu D., Grishin N.V., Chook Y.M. (2012). NESdb: A database of NES-containing CRM1 cargoes. Mol. Biol. Cell.

[76] Fu S.C., Imai K., Horton P. (2011). Prediction of leucine-rich nuclear export signal containing proteins with NESsential. Nucleic Acids. Res..

[77] Dellaire G., Farrall R., Bickmore W.A. (2003). The Nuclear Protein Database (NPD): Sub-nuclear localisation and functional annotation of the nuclear proteome. Nucleic Acids Res..

[78] Frege T., Uversky V.N. (2015). Intrinsically disordered proteins in the nucleus of human cells. Biochem. Biophys. Rep..

[79] Liu J., Perumal N.B., Oldfield C.J., Su E.W., Uversky V.N., Dunker A.K. (2006). Intrinsic disorder in transcription factors. Biochemistry.

[80] Craig E., Zhang Z.K., Davies K.P., Kalpana G.V. (2002). A masked NES in INI1/hSNF5 mediates hCRM1-dependent nuclear export: Implications for tumorigenesis. EMBO J..

[81] Romero P., Obradovic Z., Li X., Garner E.C., Brown C.J., Dunker A.K. (2001). Sequence complexity of disordered protein. Proteins.

[82] He B., Wang K., Liu Y., Xue B., Uversky V.N., Dunker A.K. (2009). Predicting intrinsic disorder in proteins: An overview. Cell Res..

[83] Das S., Cano J., Kalpana G.V. (2009). Multimerization and DNA binding properties of INI1/hSNF5 and its functional significance. J. Biol. Chem..

[84] Borg N.A. (2016). Ubiquitin Signaling to NF-Kb.

[85] Latimer M., Ernst M.K., Dunn L.L., Drutskaya M., Rice N.R. (1998). The N-terminal domain of IkappaB alpha masks the nuclear localization signal(s) of p50 and c-Rel homodimers. Mol. Cell Biol..

[86] Jacobs M.D., Harrison S.C. (1998). Structure of an IkappaBalpha/NF-kappaB complex. Cell.

[87] Muller C.W., Rey F.A., Sodeoka M., Verdine G.L., Harrison S.C. (1995). Structure of the NF-kappa B p50 homodimer bound to DNA. Nature.

[88] Huang D.B., Huxford T., Chen Y.Q., Ghosh G. (1997). The role of DNA in the mechanism of NFkappaB dimer formation: Crystal structures of the dimerization domains of the p50 and p65 subunits. Structure.

[89] Ghosh G., van Duyne G., Ghosh S., Sigler P.B. (1995). Structure of NF-kappa B p50 homodimer bound to a kappa B site. Nature.

[90] Westerheide S.D., Raynes R., Powell C., Xue B., Uversky V.N. (2012). HSF transcription factor family, heat shock response, and protein intrinsic disorder. Curr. Protein Pept. Sci..

[91] Hatos A., Hajdu-Soltesz B., Monzon A.M., Palopoli N., Alvarez L., Aykac-Fas B., Bassot C., Benitez G.I., Bevilacqua M., Chasapi A. (2020). DisProt: Intrinsic protein disorder annotation in 2020. Nucleic Acids Res..

[92] Goh G.K., Dunker A.K., Uversky V.N. (2016). Correlating Flavivirus virulence and levels of intrinsic disorder in shell proteins: Protective roles vs. immune evasion. Mol. Biosyst..

[93] Goh G.K., Dunker A.K., Uversky V.N. (2009). Protein intrinsic disorder and influenza virulence: The 1918 H1N1 and H5N1 viruses. Virol. J..

[94] Goh G.K., Dunker A.K., Foster J.A., Uversky V.N. (2020). Shell Disorder Analysis Suggests That Pangolins Offered a Window for a Silent Spread of an Attenuated SARS-CoV-2 Precursor among Humans. J. Proteome Res..

[95] Goh G.K., Dunker A.K., Foster J.A., Uversky V.N. (2020). Nipah shell disorder, modes of infection, and virulence. Microb. Pathog..

[96] Goh G.K., Dunker A.K., Foster J.A., Uversky V.N. (2019). Zika and Flavivirus Shell Disorder: Virulence and Fetal Morbidity. Biomolecules.

[97] Cohen S., Au S., Pante N. (2011). How viruses access the nucleus. Biochim. Biophys. Acta.

[98] Yarbrough M.L., Mata M.A., Sakthivel R., Fontoura B.M. (2014). Viral subversion of nucleocytoplasmic trafficking. Traffic.

[99] Le Sage V., Mouland A.J. (2013). Viral subversion of the nuclear pore complex. Viruses.

[100] Trotman L.C., Mosberger N., Fornerod M., Stidwill R.P., Greber U.F. (2001). Import of adenovirus Dann involves the nuclear pore complex receptor CAN/Nup214 and histone H1. Nat. Cell Biol..

[101] Cassany A., Ragues J., Guan T., Begu D., Wodrich H., Kann M., Nemerow G.R., Gerace L. (2015). Nuclear import of adenovirus DNA involves direct interaction of hexon with an N-terminal domain of the nucleoporin Nup214. J. Virol..

[102] Strunze S., Engelke M.F., Wang I.H., Puntener D., Boucke K., Schleich S., Way M., Schoenenberger P., Burckhardt C.J., Greber U.F. (2011). Kinesin-1-mediated capsid disassembly and disruption of the nuclear pore complex promote virus infection. Cell Host Microbe.

[103] Wodrich H., Cassany A., D’Angelo M.A., Guan T., Nemerow G., Gerace L. (2006). Adenovirus core protein pVII is translocated into the nucleus by multiple import receptor pathways. J. Virol..

[104] De Jesus-Gonzalez L.A., Cervantes-Salazar M., Reyes-Ruiz J.M., Osuna-Ramos J.F., Farfan-Morales C.N., Palacios-Rapalo S.N., Perez-Olais J.H., Cordero-Rivera C.D., Hurtado-Monzon A.M., Ruiz-Jimenez F. (2020). The Nuclear Pore Complex: A Target for NS3 Protease of Dengue and Zika Viruses. Viruses.

[105] Watters K., Palmenberg A.C. (2011). Differential processing of nuclear pore complex proteins by rhinovirus 2A proteases from different species and serotypes. J. Virol..

[106] Park Y.C., Burkitt V., Villa A.R., Tong L., Wu H. (1999). Structural basis for self-association and receptor recognition of human TRAF2. Nature.

[107] Park N., Skern T., Gustin K.E. (2010). Specific cleavage of the nuclear pore complex protein Nup62 by a viral protease. J. Biol. Chem..

[108] Park N., Katikaneni P., Skern T., Gustin K.E. (2008). Differential targeting of nuclear pore complex proteins in poliovirus-infected cells. J. Virol..

[109] Ghildyal R., Jordan B., Li D., Dagher H., Bardin P.G., Gern J.E., Jans D.A. (2009). Rhinovirus 3C protease can localize in the nucleus and alter active and passive nucleocytoplasmic transport. J. Virol..

[110] Gustin K.E., Sarnow P. (2002). Inhibition of nuclear import and alteration of nuclear pore complex composition by rhinovirus. J. Virol..

[111] Gustin K.E., Sarnow P. (2001). Effects of poliovirus infection on nucleo-cytoplasmic trafficking and nuclear pore complex composition. EMBO J..

[112] Castello A., Izquierdo J.M., Welnowska E., Carrasco L. (2009). RNA nuclear export is blocked by poliovirus 2A protease and is concomitant with nucleoporin cleavage. J. Cell Sci..

[113] Park N., Schweers N.J., Gustin K.E. (2015). Selective Removal of FG Repeat Domains from the Nuclear Pore Complex by Enterovirus 2A(pro). J. Virol..

[114] Belov G.A., Lidsky P.V., Mikitas O.V., Egger D., Lukyanov K.A., Bienz K., Agol V.I. (2004). Bidirectional increase in permeability of nuclear envelope upon poliovirus infection and accompanying alterations of nuclear pores. J. Virol..

[115] Oberste M.S., Gotuzzo E., Blair P., Nix W.A., Ksiazek T.G., Comer J.A., Rollin P., Goldsmith C.S., Olson J., Kochel T.J. (2009). Human febrile illness caused by encephalomyocarditis virus infection, Peru. Emerg. Infect. Dis..

[116] Czechowicz J., Huaman J.L., Forshey B.M., Morrison A.C., Castillo R., Huaman A., Caceda R., Eza D., Rocha C., Blair P.J. (2011). Prevalence and risk factors for encephalomyocarditis virus infection in Peru. Vector Borne Zoonotic Dis..

[117] Porter F.W., Bochkov Y.A., Albee A.J., Wiese C., Palmenberg A.C. (2006). A picornavirus protein interacts with Ran-GTPase and disrupts nucleocytoplasmic transport. Proc. Natl. Acad. Sci. USA.

[118] Porter F.W., Palmenberg A.C. (2009). Leader-induced phosphorylation of nucleoporins correlates with nuclear trafficking inhibition by cardioviruses. J. Virol..

[119] Bardina M.V., Lidsky P.V., Sheval E.V., Fominykh K.V., van Kuppeveld F.J., Polyakov V.Y., Agol V.I. (2009). Mengovirus-induced rearrangement of the nuclear pore complex: Hijacking cellular phosphorylation machinery. J. Virol..

[120] Ciomperlik J.J., Basta H.A., Palmenberg A.C. (2015). Three cardiovirus Leader proteins equivalently inhibit four different nucleocytoplasmic trafficking pathways. Virology.

[121] Lidsky P.V., Hato S., Bardina M.V., Aminev A.G., Palmenberg A.C., Sheval E.V., Polyakov V.Y., van Kuppeveld F.J., Agol V.I. (2006). Nucleocytoplasmic traffic disorder induced by cardioviruses. J. Virol..

[122] Shindo Y., Iwamoto K., Mouri K., Hibino K., Tomita M., Kosako H., Sako Y., Takahashi K. (2016). Conversion of graded phosphorylation into switch-like nuclear translocation via autoregulatory mechanisms in ERK signalling. Nat. Commun..

[123] Kehlenbach R.H., Gerace L. (2000). Phosphorylation of the nuclear transport machinery down-regulates nuclear protein import in vitro. J. Biol. Chem..

[124] Mishra A., Sipma W., Veenhoff L.M., Van der Giessen E., Onck P.R. (2019). The Effect of FG-Nup Phosphorylation on NPC Selectivity: A One-Bead-Per-Amino-Acid Molecular Dynamics Study. Int. J. Mol. Sci..

[125] Neufeldt C.J., Joyce M.A., Levin A., Steenbergen R.H., Pang D., Shields J., Tyrrell D.L., Wozniak R.W. (2013). Hepatitis C virus-induced cytoplasmic organelles use the nuclear transport machinery to establish an environment conducive to virus replication. PLoS Pathog..

[126] Levin A., Neufeldt C.J., Pang D., Wilson K., Loewen-Dobler D., Joyce M.A., Wozniak R.W., Tyrrell D.L. (2014). Functional characterization of nuclear localization and export signals in hepatitis C virus proteins and their role in the membranous web. PLoS ONE.

[127] Henderson B.R., Percipalle P. (1997). Interactions between HIV Rev and nuclear import and export factors: The Rev nuclear localisation signal mediates specific binding to human importin-beta. J. Mol. Biol..

[128] Wang P., Palese P., O’Neill R.E. (1997). The NPI-1/NPI-3 (karyopherin alpha) binding site on the influenza a virus nucleoprotein NP is a nonconventional nuclear localization signal. J. Virol..

[129] Weber F., Kochs G., Gruber S., Haller O. (1998). A classical bipartite nuclear localization signal on Thogoto and influenza A virus nucleoproteins. Virology.

[130] Atkinson S.C., Audsley M.D., Lieu K.G., Marsh G.A., Thomas D.R., Heaton S.M., Paxman J.J., Wagstaff K.M., Buckle A.M., Moseley G.W. (2018). Recognition by host nuclear transport proteins drives disorder-to-order transition in Hendra virus V. Sci. Rep..

[131] Ghildyal R., Ho A., Dias M., Soegiyono L., Bardin P.G., Tran K.C., Teng M.N., Jans D.A. (2009). The respiratory syncytial virus matrix protein possesses a Crm1-mediated nuclear export mechanism. J. Virol..

[132] Huang S., Chen J., Chen Q., Wang H., Yao Y., Chen J., Chen Z. (2013). A second CRM1-dependent nuclear export signal in the influenza A virus NS2 protein contributes to the nuclear export of viral ribonucleoproteins. J. Virol..

[133] Anderson G., Wang R., Bandyopadhyay A., Goodin M. (2012). The Nucleocapsid Protein of Potato Yellow dwarf Virus: Protein Interactions and Nuclear Import Mediated by a Non-Canonical Nuclear Localization Signal. Front. Plant. Sci..

[134] Fernandez J., Machado A.K., Lyonnais S., Chamontin C., Gartner K., Leger T., Henriquet C., Garcia C., Portilho D.M., Pugniere M. (2019). Transportin-1 binds to the HIV-1 capsid via a nuclear localization signal and triggers uncoating. Nat. Microbiol..

[135] Sparrer K.M., Pfaller C.K., Conzelmann K.K. (2012). Measles virus C protein interferes with Beta interferon transcription in the nucleus. J. Virol..

[136] Kobayashi T., Kamitani W., Zhang G., Watanabe M., Tomonaga K., Ikuta K. (2001). Borna disease virus nucleoprotein requires both nuclear localization and export activities for viral nucleocytoplasmic shuttling. J. Virol..

[137] Kim F.J., Beeche A.A., Hunter J.J., Chin D.J., Hope T.J. (1996). Characterization of the nuclear export signal of human T-cell lymphotropic virus type 1 Rex reveals that nuclear export is mediated by position-variable hydrophobic interactions. Mol. Cell Biol..

[138] Siomi H., Shida H., Nam S.H., Nosaka T., Maki M., Hatanaka M. (1988). Sequence requirements for nucleolar localization of human T cell leukemia virus type I pX protein, which regulates viral RNA processing. Cell.

[139] DiMattia M.A., Watts N.R., Stahl S.J., Rader C., Wingfield P.T., Stuart D.I., Steven A.C., Grimes J.M. (2010). Implications of the HIV-1 Rev dimer structure at 3.2 A resolution for multimeric binding to the Rev response element. Proc. Natl. Acad. Sci. USA.

[140] Daugherty M.D., Liu B., Frankel A.D. (2010). Structural basis for cooperative RNA binding and export complex assembly by HIV Rev. Nat. Struct. Mol. Biol..

[141] Hutten S., Walde S., Spillner C., Hauber J., Kehlenbach R.H. (2009). The nuclear pore component Nup358 promotes transportin-dependent nuclear import. J. Cell Sci..

[142] Gu L., Tsuji T., Jarboui M.A., Yeo G.P., Sheehy N., Hall W.W., Gautier V.W. (2011). Intermolecular masking of the HIV-1 Rev NLS by the cellular protein HIC: Novel insights into the regulation of Rev nuclear import. Retrovirology.

[143] Arnold M., Nath A., Hauber J., Kehlenbach R.H. (2006). Multiple importins function as nuclear transport receptors for the Rev protein of human immunodeficiency virus type 1. J. Biol. Chem..

[144] Ye X., Gorin A., Frederick R., Hu W., Majumdar A., Xu W., McLendon G., Ellington A., Patel D.J. (1999). RNA architecture dictates the conformations of a bound peptide. Chem. Biol..

[145] Casu F., Duggan B.M., Hennig M. (2013). The arginine-rich RNA-binding motif of HIV-1 Rev is intrinsically disordered and folds upon RRE binding. Biophys. J..

[146] Daugherty M.D., Booth D.S., Jayaraman B., Cheng Y., Frankel A.D. (2010). HIV Rev response element (RRE) directs assembly of the Rev homooligomer into discrete asymmetric complexes. Proc. Natl. Acad. Sci. USA.

[147] Auer M., Gremlich H.U., Seifert J.M., Daly T.J., Parslow T.G., Casari G., Gstach H. (1994). Helix-loop-helix motif in HIV-1 Rev. Biochemistry.

[148] Behrens R.T., Aligeti M., Pocock G.M., Higgins C.A., Sherer N.M. (2017). Nuclear Export Signal Masking Regulates HIV-1 Rev Trafficking and Viral RNA Nuclear Export. J. Virol..

[149] Fineberg K., Fineberg T., Graessmann A., Luedtke N.W., Tor Y., Lixin R., Jans D.A., Loyter A. (2003). Inhibition of nuclear import mediated by the Rev-arginine rich motif by RNA molecules. Biochemistry.

[150] Malim M.H., Cullen B.R. (1991). HIV-1 structural gene expression requires the binding of multiple Rev monomers to the viral RRE: Implications for HIV-1 latency. Cell.

[151] Faust O., Grunhaus D., Shimshon O., Yavin E., Friedler A. (2018). Protein Regulation by Intrinsically Disordered Regions: A Role for Subdomains in the IDR of the HIV-1 Rev Protein. Chembiochem.

[152] Jayaraman B., Fernandes J.D., Yang S., Smith C., Frankel A.D. (2019). Highly Mutable Linker Regions Regulate HIV-1 Rev Function and Stability. Sci. Rep..

[153] Suzuki R., Matsuura Y., Suzuki T., Ando A., Chiba J., Harada S., Saito I., Miyamura T. (1995). Nuclear localization of the truncated hepatitis C virus core protein with its hydrophobic C terminus deleted. J. Gen. Virol..

[154] Song J., Nagano-Fujii M., Wang F., Florese R., Fujita T., Ishido S., Hotta H. (2000). Nuclear localization and intramolecular cleavage of N-terminally deleted NS5A protein of hepatitis C virus. Virus Res..

[155] Kunkel M., Watowich S.J. (2004). Biophysical characterization of hepatitis C virus core protein: Implications for interactions within the virus and host. FEBS Lett..

[156] Boulant S., Vanbelle C., Ebel C., Penin F., Lavergne J.P. (2005). Hepatitis C virus core protein is a dimeric alpha-helical protein exhibiting membrane protein features. J. Virol..

[157] Duvignaud J.B., Savard C., Fromentin R., Majeau N., Leclerc D., Gagne S.M. (2009). Structure and dynamics of the N-terminal half of hepatitis C virus core protein: An intrinsically unstructured protein. Biochem. Biophys. Res. Commun..

[158] Shaw M.L., Cardenas W.B., Zamarin D., Palese P., Basler C.F. (2005). Nuclear localization of the Nipah virus W protein allows for inhibition of both virus- and toll-like receptor 3-triggered signaling pathways. J. Virol..

[159] Audsley M.D., Jans D.A., Moseley G.W. (2016). Nucleocytoplasmic trafficking of Nipah virus W protein involves multiple discrete interactions with the nuclear import and export machinery. Biochem. Biophys. Res. Commun..

[160] Xiao C.Y., Hubner S., Elliot R.M., Caon A., Jans D.A. (1996). A consensus cAMP-dependent protein kinase (PK-A) site in place of the CcN motif casein kinase II site simian virus 40 large T-antigen confers PK-A-mediated regulation of nuclear import. J. Biol. Chem..

[161] Hubner S., Xiao C.Y., Jans D.A. (1997). The protein kinase CK2 site (Ser111/112) enhances recognition of the simian virus 40 large T-antigen nuclear localization sequence by importin. J. Biol. Chem..

[162] Yang S.N.Y., Atkinson S.C., Wang C., Lee A., Bogoyevitch M.A., Borg N.A., Jans D.A. (2020). The broad spectrum antiviral ivermectin targets the host nuclear transport importin alpha/beta1 heterodimer. Antiviral Res..

[163] King C.R., Tessier T.M., Dodge M.J., Weinberg J.B., Mymryk J.S. (2020). Inhibition of Human Adenovirus Replication by the Importin alpha/beta1 Nuclear Import Inhibitor Ivermectin. J. Virol..

[164] Kosyna F.K., Nagel M., Kluxen L., Kraushaar K., Depping R. (2015). The importin alpha/beta-specific inhibitor Ivermectin affects HIF-dependent hypoxia response pathways. Biol. Chem..

[165] Fraser J.E., Rawlinson S.M., Wang C., Jans D.A., Wagstaff K.M. (2014). Investigating dengue virus nonstructural protein 5 (NS5) nuclear import. Methods Mol. Biol..

[166] Lv C., Liu W., Wang B., Dang R., Qiu L., Ren J., Yan C., Yang Z., Wang X. (2018). Ivermectin inhibits DNA polymerase UL42 of pseudorabies virus entrance into the nucleus and proliferation of the virus in vitro and vivo. Antiviral Res..

[167] Raza S., Shahin F., Zhai W., Li H., Alvisi G., Yang K., Chen X., Chen Y., Chen J., Hu C. (2020). Ivermectin Inhibits Bovine Herpesvirus 1 DNA Polymerase Nuclear Import and Interferes with Viral Replication. Microorganisms.

[168] Mastrangelo E., Pezzullo M., De Burghgraeve T., Kaptein S., Pastorino B., Dallmeier K., de Lamballerie X., Neyts J., Hanson A.M., Frick D.N. (2012). Ivermectin is a potent inhibitor of flavivirus replication specifically targeting NS3 helicase activity: New prospects for an old drug. J. Antimicrob. Chemother..

[169] Lundberg L., Pinkham C., Baer A., Amaya M., Narayanan A., Wagstaff K.M., Jans D.A., Kehn-Hall K. (2013). Nuclear import and export inhibitors alter capsid protein distribution in mammalian cells and reduce Venezuelan Equine Encephalitis Virus replication. Antiviral Res..

[170] Barrows N.J., Campos R.K., Powell S.T., Prasanth K.R., Schott-Lerner G., Soto-Acosta R., Galarza-Munoz G., McGrath E.L., Urrabaz-Garza R., Gao J. (2016). A Screen of FDA-Approved Drugs for Inhibitors of Zika Virus Infection. Cell Host Microbe.

[171] Wagstaff K.M., Sivakumaran H., Heaton S.M., Harrich D., Jans D.A. (2012). Ivermectin is a specific inhibitor of importin alpha/beta-mediated nuclear import able to inhibit replication of HIV-1 and dengue virus. Biochem. J..

[172] Fraser J.E., Watanabe S., Wang C., Chan W.K., Maher B., Lopez-Denman A., Hick C., Wagstaff K.M., Mackenzie J.M., Sexton P.M. (2014). A nuclear transport inhibitor that modulates the unfolded protein response and provides in vivo protection against lethal dengue virus infection. J. Infect. Dis..

[173] Tay M.Y., Fraser J.E., Chan W.K., Moreland N.J., Rathore A.P., Wang C., Vasudevan S.G., Jans D.A. (2013). Nuclear localization of dengue virus (DENV) 1-4 non-structural protein 5; protection against all 4 DENV serotypes by the inhibitor Ivermectin. Antiviral Res..

[174] Gotz V., Magar L., Dornfeld D., Giese S., Pohlmann A., Hoper D., Kong B.W., Jans D.A., Beer M., Haller O. (2016). Influenza A viruses escape from MxA restriction at the expense of efficient nuclear vRNP import. Sci. Rep..

[175] Caly L., Druce J.D., Catton M.G., Jans D.A., Wagstaff K.M. (2020). The FDA-approved drug ivermectin inhibits the replication of SARS-CoV-2 in vitro. Antiviral Res..

[176] Ketkar H., Yang L., Wormser G.P., Wang P. (2019). Lack of efficacy of ivermectin for prevention of a lethal Zika virus infection in a murine system. Diagn. Microbiol. Infect. Dis..

[177] Hintersteiner M., Ambrus G., Bednenko J., Schmied M., Knox A.J., Meisner N.C., Gstach H., Seifert J.M., Singer E.L., Gerace L. (2010). Identification of a small molecule inhibitor of importin beta mediated nuclear import by confocal on-bead screening of tagged one-bead one-compound libraries. ACS Chem. Biol..

[178] Soderholm J.F., Bird S.L., Kalab P., Sampathkumar Y., Hasegawa K., Uehara-Bingen M., Weis K., Heald R. (2011). Importazole, a small molecule inhibitor of the transport receptor importin-beta. ACS Chem. Biol..

[179] Sun Q., Carrasco Y.P., Hu Y., Guo X., Mirzaei H., Macmillan J., Chook Y.M. (2013). Nuclear export inhibition through covalent conjugation and hydrolysis of Leptomycin B by CRM1. Proc. Natl. Acad. Sci. USA.

[180] Wolff B., Sanglier J.J., Wang Y. (1997). Leptomycin B is an inhibitor of nuclear export: Inhibition of nucleo-cytoplasmic translocation of the human immunodeficiency virus type 1 (HIV-1) Rev protein and Rev-dependent mRNA. Chem. Biol..

[181] Elton D., Simpson-Holley M., Archer K., Medcalf L., Hallam R., McCauley J., Digard P. (2001). Interaction of the influenza virus nucleoprotein with the cellular CRM1-mediated nuclear export pathway. J. Virol..

[182] Newlands E.S., Rustin G.J., Brampton M.H. (1996). Phase I trial of elactocin. Br. J. Cancer.

[183] Hing Z.A., Fung H.Y., Ranganathan P., Mitchell S., El-Gamal D., Woyach J.A., Williams K., Goettl V.M., Smith J., Yu X. (2016). Next-generation XPO1 inhibitor shows improved efficacy and in vivo tolerability in hematological malignancies. Leukemia.

[184] Etchin J., Berezovskaya A., Conway A.S., Galinsky I.A., Stone R.M., Baloglu E., Senapedis W., Landesman Y., Kauffman M., Shacham S. (2017). KPT-8602, a second-generation inhibitor of XPO1-mediated nuclear export, is well tolerated and highly active against AML blasts and leukemia-initiating cells. Leukemia.

[185] Neira J.L., Bintz J., Arruebo M., Rizzuti B., Bonacci T., Vega S., Lanas A., Velazquez-Campoy A., Iovanna J.L., Abian O. (2017). Identification of a Drug Targeting an Intrinsically Disordered Protein Involved in Pancreatic Adenocarcinoma. Sci. Rep..

[186] Iconaru L.I., Ban D., Bharatham K., Ramanathan A., Zhang W., Shelat A.A., Zuo J., Kriwacki R.W. (2015). Discovery of Small Molecules that Inhibit the Disordered Protein, p27(Kip1). Sci. Rep..

[187] Hammoudeh D.I., Follis A.V., Prochownik E.V., Metallo S.J. (2009). Multiple independent binding sites for small-molecule inhibitors on the oncoprotein c-Myc. J. Am. Chem. Soc..

[188] Follis A.V., Hammoudeh D.I., Wang H., Prochownik E.V., Metallo S.J. (2008). Structural rationale for the coupled binding and unfolding of the c-Myc oncoprotein by small molecules. Chem. Biol..

